# Minimizing the Risk of Disease Transmission in Emergency Settings: Novel *In Situ* Physico-Chemical Disinfection of Pathogen-Laden Hospital Wastewaters

**DOI:** 10.1371/journal.pntd.0003776

**Published:** 2015-06-25

**Authors:** Emanuele Sozzi, Kerline Fabre, Jean-François Fesselet, James E. Ebdon, Huw Taylor

**Affiliations:** 1 School of Environment and Technology, University of Brighton, Brighton, United Kingdom; 2 MSF-OCA, Port-au-Prince, Haiti; 3 MSF-OCA, Amsterdam, Holland; Universidad Peruana Cayetano Heredia, PERU

## Abstract

The operation of a health care facility, such as a cholera or Ebola treatment center in an emergency setting, results in the production of pathogen-laden wastewaters that may potentially lead to onward transmission of the disease. The research presented here evaluated the design and operation of a novel treatment system, successfully used by *Médecins Sans Frontières* in Haiti to disinfect CTC wastewaters *in situ*, eliminating the need for road haulage and disposal of the waste to a poorly-managed hazardous waste facility, thereby providing an effective barrier to disease transmission through a novel but simple sanitary intervention. The physico-chemical protocols eventually successfully treated over 600 m^3^ of wastewater, achieving coagulation/flocculation and disinfection by exposure to high pH (Protocol A) and low pH (Protocol B) environments, using thermotolerant coliforms as a disinfection efficacy index. In Protocol A, the addition of hydrated lime resulted in wastewater disinfection and coagulation/flocculation of suspended solids. In Protocol B, disinfection was achieved by the addition of hydrochloric acid, followed by pH neutralization and coagulation/flocculation of suspended solids using aluminum sulfate. Removal rates achieved were: COD >99%; suspended solids >90%; turbidity >90% and thermotolerant coliforms >99.9%. The proposed approach is the first known successful attempt to disinfect wastewater in a disease outbreak setting without resorting to the alternative, untested, approach of ‘super chlorination’ which, it has been suggested, may not consistently achieve adequate disinfection. A basic analysis of costs demonstrated a significant saving in reagent costs compared with the less reliable approach of super-chlorination. The proposed approach to *in situ* sanitation in cholera treatment centers and other disease outbreak settings represents a timely response to a UN call for onsite disinfection of wastewaters generated in such emergencies, and the ‘Coalition for Cholera Prevention and Control’ recently highlighted the research as meriting serious consideration and further study. Further applications of the method to other emergency settings are being actively explored by the authors through discussion with the World Health Organization with regards to the ongoing Ebola outbreak in West Africa, and with the UK-based NGO Oxfam with regards to excreta-borne disease management in the Philippines and Myanmar, as a component of post-disaster incremental improvements to local sanitation chains.

## Author Summary

When an outbreak of infectious disease occurs in a low-resource setting, the rapid construction of emergency healthcare facilities may significantly reduce mortality. The facilities also result in the generation of large volumes of highly contaminated fecal waste that represents a potential basis for further disease transmission. Infection protection and control strategies at healthcare facilities must therefore include measures to establish and maintain good water supplies, sanitation and hygiene (WASH). Even where the pathogen of concern is not *waterborne*, health-care providers have a ‘duty-of-care’ to protect workers and neighboring communities from all excreta-borne diseases. In this study, the authors successfully demonstrated, for the first time, the *in situ* disinfection of wastewaters from cholera treatment centers during the Haiti cholera outbreak, using a low-cost physicochemical method. The approach is currently being adapted by NGOs to help manage human excreta in other emergency settings, including the current Ebola outbreak. Although the Ebola virus is relatively fragile, it may exist in high concentrations in the bodily fluids (including feces) of those with the disease. The approach to *in situ* disinfection of excreta described here may therefore support infection control in outbreaks of Ebola and other infectious diseases.

## Introduction

Outbreaks of specific infectious diseases that may potentially be transmitted by human excreta, including cholera, Ebola and hepatitis A and E present a challenge to existing WASH (water, sanitation and hygiene) practices and a greater focus on practical *in situ* disinfection of human waste may offer an effective first step in the development of a longer-term sanitation ladder to support infection control. The research presented here focuses on an innovative *in situ* disinfection technique, which to date has been mainly applied in the context of a cholera outbreak, but which could potentially, and in the near future, provide a health protection intervention within the context of other outbreaks of neglected tropical diseases, including Ebola.

Ten months after the devastating earthquake of 12^th^ January 2010, cholera appeared in Haiti for the first time in nearly a century. The outbreak escalated and as of 21^st^ November 2014, the resulting mortality had reached 8,505 and the cumulative morbidity had reached 717,203—equivalent to approximately 6.9 percent of the national population [1] [2]. According to the WHO [3], the outbreak accounted for 57% and 53% of global cholera cases, and 58% and 37% of global cholera deaths reported in 2010 and 2011 respectively. Morbidity levels have probably been significantly higher than these figures suggest, as globally only a minority of cholera cases may be reported to the relevant authorities [3].

Cholera is a severe, acute, dehydrating diarrheal disease of humans, which, in the absence of adequate rehydration, can lead to death in both children and adults within twelve hours. The case-fatality rate for severe cholera without treatment can be as high as 50% [4]. The disease results from infection by a pathogenic strain of the bacterium *Vibrio cholerae*, which is capable of producing a potent toxin. Since the first recorded cholera pandemic, which began in 1816, the pathogen has spread and evolved rapidly [4] [5]. The ongoing seventh cholera pandemic began in 1961 and there is now good molecular evidence to suggest a close relationship between the Haitian isolates of *V*. *cholerae* and variant *V*. *cholerae* El Tor O1 strains isolated in Bangladesh in 2002 and 2008, and a more distant relationship with isolates currently circulating in South America [6] [7].

Established cholera control strategies call for a combination of interventions, including improvements to the quality and quantity of drinking water supplies, provision of consistently functional sanitation chains and promotion of effective hygiene practices. Under certain circumstances, the administration of oral vaccines to ‘at risk’ communities may also be recommended [8] [9]. Treatment of infected individuals is largely based on oral (or in more serious cases, intravenous) rehydration [4]. For the most severe cases, a suitable antibiotic, such as tetracycline, doxycycline or azithromycin, may be administered [10]. However, it has been widely recognized that treatment alone will not break the cycle of disease transmission and that improvements of WASH infrastructure are essential to achieving sustained control, elimination, or eradication of many tropical diseases [11] [12] [13] [14].

*Doctors Without Borders* (*Médecins Sans Frontières*, or MSF) is an international medical humanitarian organization that delivers emergency aid to people affected by armed conflict, epidemics, natural disasters, and exclusion from healthcare. It has operated successfully in numerous cholera emergencies during the past four decades, and has formulated effective field response strategies to cholera outbreaks, including the design and operation of appropriate water and sanitation technologies. The organization has been active in Haiti for over twenty years and, in collaboration with the Haitian Ministry of Public Health (MSPP), has been a leading provider of treatment to cholera patients in the country since the beginning of the outbreak, treating more than 300,000 patients by September 2014 [15] [16] [17].

The established MSF protocol for dealing with human fecal wastes in emergencies involves the addition of 2% chlorine solution to each bucket of patient feces or vomit and the construction and operation of soil infiltration pits or trenches to dispose of the large volumes of waste produced by CTC operations [18]. However, this approach is only appropriate when the water table remains at least 1.5 meters below the lowest point of the excavated pit or trench. In the densely populated Haitian capital of Port-au-Prince, the water table may be considerably higher (as little as 30 cm below the surface during periods of heavy rainfall), and clearly, under these circumstances, infiltration cannot provide safe disposal of the infectious human wastes arising [19].

From the outset, the response of many of the international organizations operating in the wake of the Haiti cholera outbreak was to instigate road haulage (by truck) of all fecal waste originating from cholera patients (chlorinated or otherwise) to a centralized waste pit at the Truitier landfill site on the outskirts of Port-au-Prince. This facility is situated close to the impoverished and densely populated community of Cité Soleil on the western outskirts of the capital, a few hundred meters from the coast and on the aquifer of the Cul-de-Sac plain, traditionally a source of raw drinking water for the city of Port-au-Prince [20]. At an early stage of the emergency response, water and sanitation engineers of MSF-OCA (*Médecins Sans Frontières*–Operational Centre Amsterdam) concluded that using the Truitier landfill site for the disposal of cholera wastes represented a clear hazard to human health and the organization therefore decided that it was not prepared to countenance this practice. The organization further decided that the practice of ‘super-chlorination’ followed by disposal to the environment was also unacceptable.

The principal reasons for these decisions were:

1. Road transportation of significant quantities of contaminated wastewater was considered hazardous to human health, particularly within the complex, often chaotic, urban context of post-emergency Port-au-Prince.
2. The contaminated wastewater arising from CTC is characterized by extremely high concentrations of readily-oxidizable matter. It would therefore be imprudent to assume that a wastewater disinfection process based on chlorination would consistently disinfect the waste to an adequate degree [21], given that the ability of these *in situ* disinfection strategies to reduce target pathogens had not been formally assessed [19].
3. It has been suggested that certain strains of *V*. *cholerae* (the “rugose” phenotype) may be more resistant to chlorine-based disinfection as a result of exopolysaccharide production, which promotes cell aggregation. Such strains may therefore pose an elevated risk to human health [22] [23].
4. Even if ‘super-chlorination’ were able to reduce *Vibrio cholerae* numbers to levels that did not pose a significant risk to those living downstream of CTC operations, the production of combined chlorine residuals and the relatively high operational costs associated with this process would likely make it both environmentally and financially unacceptable in the medium- to long-term. Moreover, this approach to disinfection does not significantly remove suspended material.

By October 2010, the rapid spread of the Haitian cholera outbreak had resulted in a pressing need for CTC facilities throughout the country and a novel, low-cost and consistently-effective way to treat and disinfect the wastewaters from MSF CTC operations was therefore urgently required.

In Port-au-Prince, a partly-commissioned MSF maternity hospital (‘Delmas 33’) was converted by the organization into a CTC within a matter of days. By the time its operational life ceased in early March 2011, more than 3,000 cholera in-patients had been treated at the facility. It was then converted back into a maternity hospital, and a new MSF CTC was established on nearby tennis courts. In total, at these two CTC, MSF water and sanitation engineers were required to treat and dispose safely of over 320,000 liters of wastewaters, which were potentially infected with high levels of *Vibrio cholerae*. It has been estimated that wastewaters from CTC, especially those components derived from patient stool buckets, may contain more than 10^7^ *Vibrio cholerae* per 100 ml [24] [25] [26]. Such wastes must therefore be treated and disposed of with extreme caution. Within the Haitian context, rapid intervention to provide effective disinfection of this wastewater was essential in order both to control disease transmission and to respond to the prevailing concerns of the local populace with regard to the management of cholera wastes by international organizations.

The onsite treatment of CTC wastewaters within the challenging context of medical emergencies needs to be relatively low-cost, logistically simple, rapid to deploy, immediately effective and capable of removing microbial pathogens significantly more effectively than conventional treatment technologies. Such systems have rarely been established, and no peer-reviewed literature that critically evaluates their operational performance is available. However, the concentration of *Vibrio cholerae* in CTC wastewaters and the potential risk to public health that the pathogen represents may be estimated from previous studies. During a two-year investigation of cholera carriers in the Philippines, Dizon *et al*. [27] measured the numbers of *Vibrio cholerae* per gram of feces among human populations in areas of the country in which the disease was endemic or epidemic. The feces of ‘simple carriers’ contained between 10^2^ and 10^5^ *Vibrio cholerae* per gram of feces, whereas the feces of patients presenting symptoms of ‘mild cholera’ were shown to contain between 10^6^ and 10^9^ *Vibrio cholerae* per milliliter of stool (on their first day of illness). Howard *et al*.[24] [25] examined the wastewater from a hospital operated by the UK-based NGO Oxfam in Bangladesh, which admitted between two and forty confirmed cholera cases per day. The authors recorded levels of *Vibrio cholerae* between 5 x 10^5^ and 5 x 10^7^ per 100 ml of wastewater. It is worth noting that the level of *Vibrio cholerae* was demonstrated to exceed that of thermotolerant coliforms in this instance.

In the work described here, the authors aimed to use the best available expertise to design, construct rapidly and operate an effective onsite CTC wastewater treatment system that would protect the health of the inhabitants of Port-au-Prince from the potential risk of disease associated with contaminated wastewaters. Further, it was considered essential that this innovative technology should be subjected to a robust critical risk evaluation of each stage of the project cycle. This was designed to maximize human health protection at the time of the emergency and to enable MSF and other NGO (potentially operating in other parts of the world), to gain the fullest possible benefit from the resulting evidence-base.

Based on initial estimations of *Vibrio cholerae* levels in the CTC wastewaters and with reference to the available literature, a wastewater management strategy, involving four consecutive and distinct barriers to the transmission of *Vibrio cholerae*, was proposed as follows:

1. Initial chlorination of patient feces within stool buckets immediately following collection by MSF health-care professionals, as already practiced according to MSF protocols [18] [28];
2. Storage of pooled CTC wastewaters in open tanks—in practice for up to twelve weeks (average six weeks, minimum three) at relatively high ambient temperatures—resulting in a further reduction in levels of enteric microorganisms as a result of natural biological, chemical and physical processes;
3. The design and operation of a novel batch-operated onsite wastewater treatment and disinfection plant, as described in detail below; and finally
4. Controlled effluent disposal within soil infiltration trenches according to existing MSF protocols [18].

In-house MSF water and sanitation expertise, supported by expert external advice, were used to develop a shortlist of three technologies that might meet the objective of achieving effective, robust and relatively low-cost onsite treatment of the CTC wastewaters:

1. Protocol A: Coagulation/flocculation and disinfection of the wastewater with hydrated (slaked) lime (Ca(OH)_2_) at high pH levels, using a treatment system that was based on the methodology of Taylor *et al*. [29] [30];
2. Protocol B: A novel approach involving disinfection at low pH levels using hydrochloric acid, followed by pH neutralization and subsequent coagulation/flocculation, achieved using aluminum sulfate (or an alternative low-cost coagulant); and
3. Protocol C: Septic tank treatment combined with an anaerobic filter.

Protocol C was rejected at an early stage, as it was considered that this approach would take too long to establish and would be insufficiently robust to operate effectively and reliably within an emergency setting. Subsequently, batch treatment systems based on Protocols A and B were designed, operated, and monitored within two CTC operations in Port-au-Prince, over a period of six months.

The main goal of the treatment was to achieve a level of disinfection of the highly contaminated fecal waste that was adequate to release the effluent and the sedimented sludge into the environment without introducing a new disease transmission route. Effective disinfection was achieved through the combined and simultaneous action of two mechanisms, namely:

1. The exposure of the pathogens to an alkaline (‘protocol A’) or acidic (‘protocol B’) environment, resulting in pathogen deactivation
2. The physical removal of the pathogen as a result of coagulation and flocculation and sedimentation. The sedimented sludge was subsequently treated in drying beds before incineration or controlled infiltration to soil (see details on the next section)

Gram-negative (Gram-) and Gram-positive (Gram+) bacteria are both sensitive to high pH, although Gram- bacteria (including *Vibrio cholerae*) tend to be more susceptible to high pH levels because of their relatively thin peptidoglycan layer: the Gram- layer is in fact only about 2 to 3 nm thick, whereas the Gram+ layer is about ten times thicker [31]. The peptidoglycan layer stabilizes the cytoplasmic membrane of intact bacterial cells against the pressure exerted by the cytoplasm [32]. Therefore, the thinner peptidoglycan layer associated with Gram- bacteria may less effectively prevent the cytoplasmic membrane from bursting once it is weakened by a high pH environment [33].

There are multiple hypotheses as to how strong and weak, organic and mineral acids inhibit or destroy bacteria. In general, acids have antimicrobial activity both in their undissociated and dissociated forms (although the former has a stronger antimicrobial effect) [34]. One of the prevailing hypotheses is that strong acids inhibit or destroy microorganisms by interfering with the permeability of the microbial cell membrane. The acidic solution interferes with the substrate transport and with the oxidative phosphorylation from the electron transport system. This results in the acidification of the cell content, which is considered to be the principal (but not the only) cause of inhibition and death [35]. It has also been suggested that some acids may also inhibit or kill bacteria by blocking amino-acid uptake through the membrane [36]. Moreover, some acids may enter the bacterial cells as undissociated molecules that are soluble in phospholipid membranes and then acidify the cell interior [37].

For the purposes of this study, thermotolerant coliforms were used as an index of disinfection efficacy. These bacteria primarily originate from the intestines of warm-blooded animals and are widely used as an indicator of the presence of fecal material in water. Although it was not feasible under the conditions of the study described here to enumerate the pathogen *Vibrio cholerae* directly, all available evidence suggests that for the extreme pH levels achieved during the treatment protocols described here, thermotolerant coliforms represent an acceptable conservative indicator of the presence of *Vibrio cholerae* in that they exist at high concentrations in human feces and are, like *Vibrio cholerae*, a Gram- bacterium of primarily enteric origin. Although future work on the behavior of specific pathogens to low-cost on-site disinfection processes is warranted, the authors believe that the approach taken here represents a robust approach to estimating the risk of pathogen transmission.

Another aspect of this study that was partly limited by the constraints of an emergency setting was chemical analysis of the wastewater. However, wastewater from cholera treatment centers is commonly composed of human feces and sullage (graywater) from personal washing facilities and hygiene practice. Therefore the main components of the wastewater are generally known though the alkalinity, the buffering capacity, the relative concentrations of organic matter and other components are liable to vary between CTC and with time. Several studies have previously determined the composition of wastewater derived from various infectious (including tropical) disease hospital departments [38] [39] [40] [41] [42]. However, although the quantity of alkaline or acidic solution required to achieve adequate treatment and disinfection by the protocols described here will depend on a number of wastewater characteristics (e.g., organic content and alkalinity) it is important to note that the protocols are defined by pH ‘end-points’, in that reagents are added until a prescribed pH level is reached so that variability in wastewater composition ceases to be a factor in treatment efficacy. Since this will be the case in other future applications of the protocols, the authors believe that the inability to obtain detailed data on the wastewater composition under the conditions of this study does not constitute a significant weakness in the research.

## Materials and Methods

Initial laboratory pilot-scale studies of Protocols A and B, using simple five-liter beakers, were followed by full-scale batch treatment of the wastewater using both protocols, initially at the ‘Delmas 33’ CTC. At a later stage, and following closure of this facility, a new, full-scale wastewater treatment facility was established at the nearby ‘Delmas-Tennis Court’ CTC. The results reported on this paper refer exclusively to the analysis of batches that were treated when adequate monitoring equipment had become available in the field. Protocol A was used for the treatment of two batches of wastewater and Protocol B was used for the large-scale treatment of six batches of CTC wastewater, the batch volumes being in all cases between 10 and 15 m^3^.

A detailed risk assessment was undertaken for each stage of the project. This included details of operator hygiene requirements and the appropriate use of personal protective equipment to minimize operator contact with potentially corrosive chemicals and infectious agents [43].

### Pilot-scale study of Protocol A

Simple jar-test studies, using five-liter beakers, were initially used to investigate the efficacy of Protocol A with regard to the removal of thermotolerant coliforms and suspended solids (or turbidity). At the inception of each jar-test, a small sample of untreated wastewater (approximately 30 ml) was taken and the following parameters were tested for: turbidity–recorded as nephalometric turbidity units (NTU); presumptive thermotolerant coliforms–recorded as colony-forming units (CFU) per 100 ml; pH level; and quantities of chemical reagents used–recorded as grams or milligrams per liter.

The first step of each jar-test experiment involved the step-wise addition of hydrated lime slurry (Ca(OH)_2_) to the wastewater, until the pH of the mix reached a level between 11.4 and 12.2. This was immediately followed by three minutes of ‘rapid-mixing’, followed by 15 minutes of ‘slow-mixing’ (both steps being achieved manually in the absence of a mechanical jar test-rig). The contents of the beaker were then left to settle overnight. The supernatant was subsequently removed and its pH level adjusted to approximately 7 by the addition of concentrated hydrochloric acid (HCl). At the end of each jar-test process, a small sample (approximately 50 ml) of supernatant was removed and tested for the same set of wastewater quality parameters as mentioned previously.

### Pilot-scale study of Protocol B

Jar-test studies were similarly performed in order to investigate the efficacy of Protocol B. At the inception of each jar-test, a small sample of untreated wastewater (approximately 30 ml) was tested for the same set of parameters as in Protocol A.

The first step of the jar-test experiment for Protocol B involved the addition of hydrochloric acid (HCl), at a quantity sufficient to decrease the wastewater pH to a level between 3.7 and 3.9, so as to achieve disinfection of the wastewater. This was immediately followed by ‘rapid-mixing’ for one minute. Following overnight sedimentation, the wastewater was adjusted to a pH level of approximately 7, by the addition of the hydrated lime slurry that was also used for Protocol A. At this point, another small sample (approximately 30 ml) of supernatant was removed for analysis as before.

Aluminum sulfate (75 to 150 mg/L—either as Al_2_(SO_4_)_3_ * 16H_2_O or as Al_2_(SO_4_)_3_ * 18H_2_O) was next added to the beaker as a coagulating agent, in order to achieve suspended solids removal, and consequently, to achieve a further reduction in microbial levels. The addition of aluminum sulfate was immediately followed by three minutes of rapid-mixing, followed by 15 minutes of slow-mixing.

Following the slow-mixing phase, the wastewater was allowed to settle in the five liter beaker reactor for one hour. Once again, a small sample of supernatant (approximately 30 ml) was removed for analysis, as before.

### Full-scale operation of the two protocols

**Fig 1:**
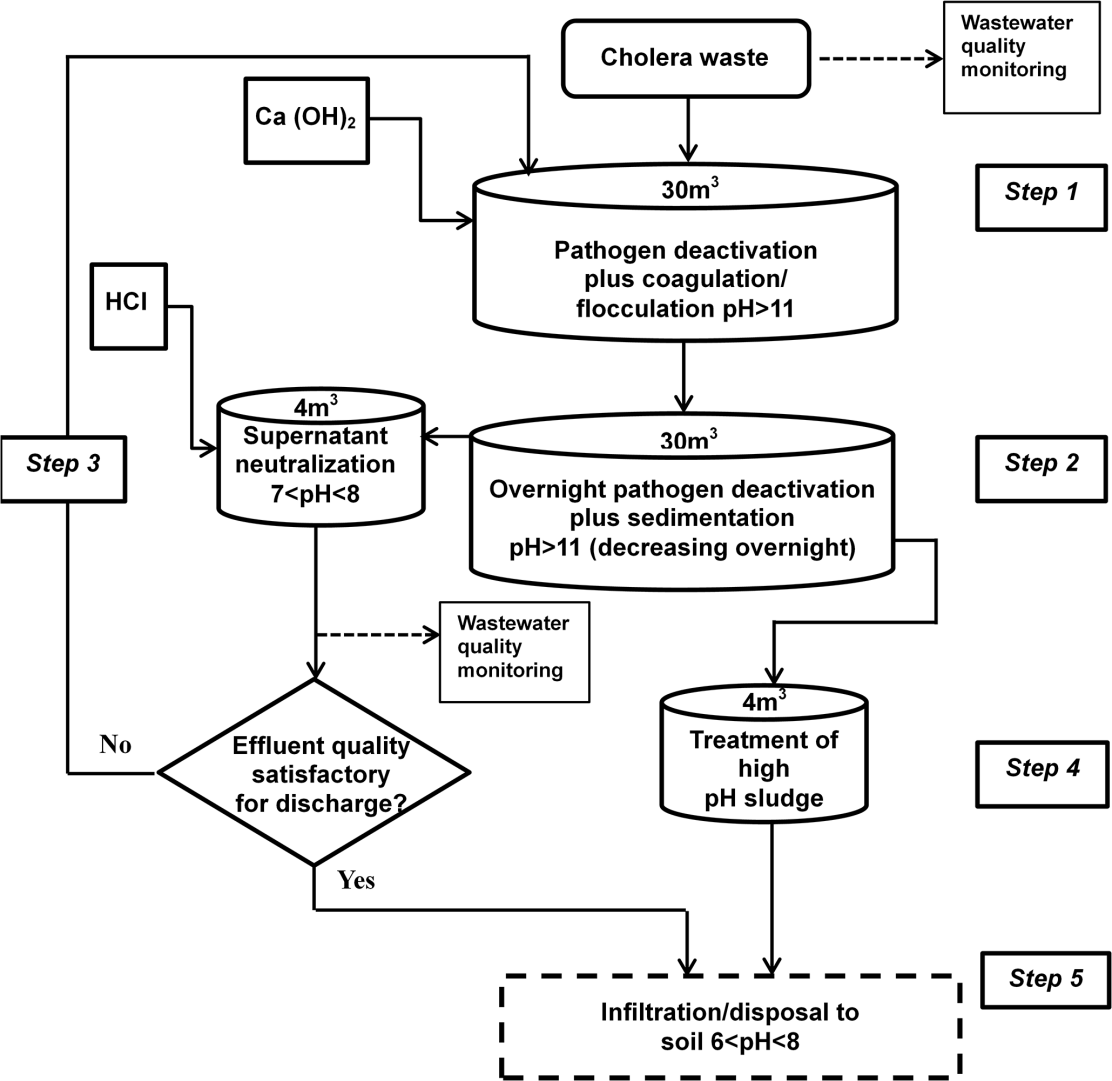
Schematic overview of the high pH (Protocol A) treatment process.

**Fig 2:**
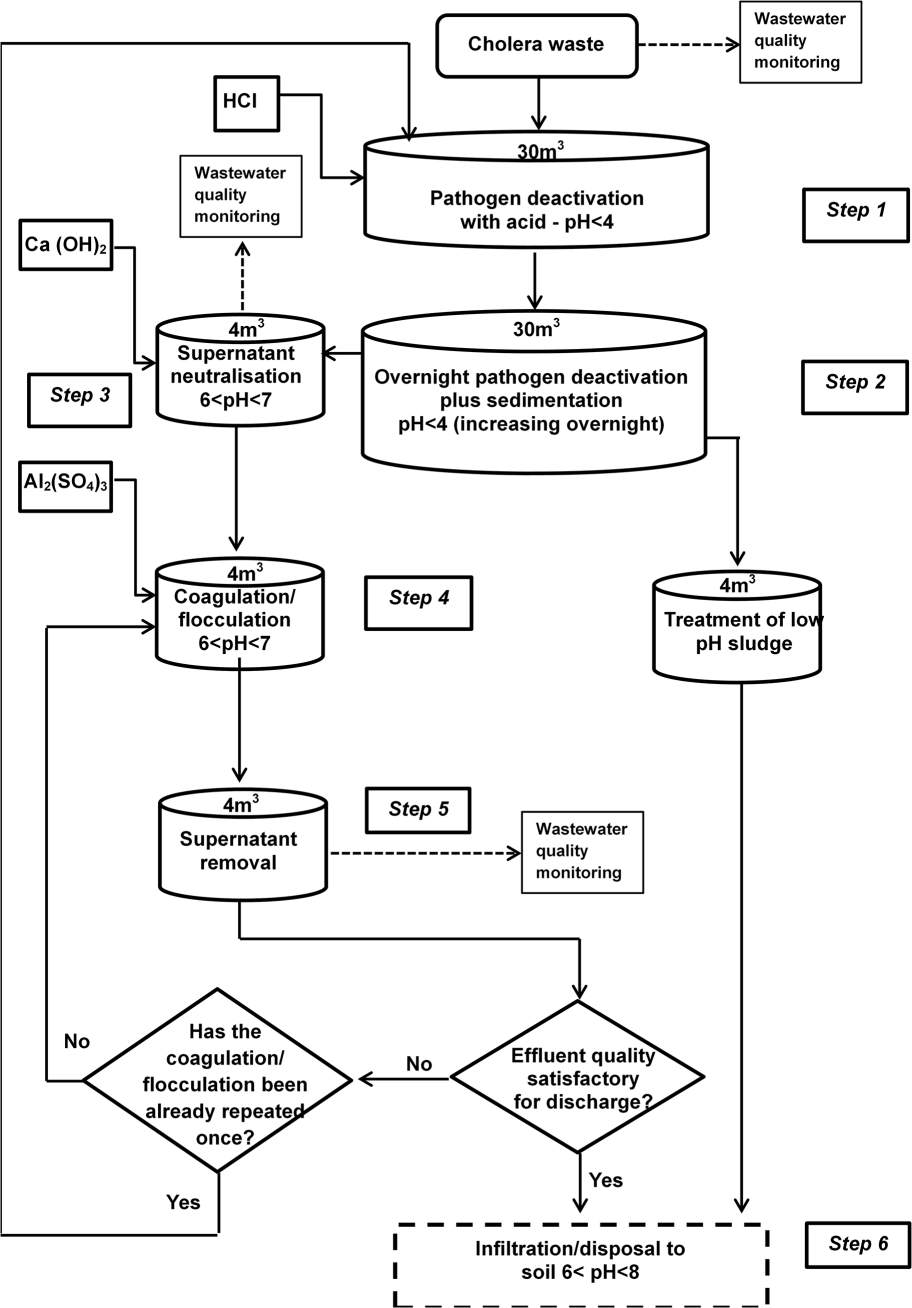
Schematic overview of the low pH (Protocol B) treatment process.

Laboratory jar-testing of the high pH treatment process (Protocol A) using hydrated lime (Ca(OH)_2_) and, at a later stage, the low pH treatment process (Protocol B) using aluminum sulfate, was followed by full-scale batch treatment. Here, wastewater and coagulants (added at concentrations suggested by the jar-tests) were combined within regimes that mimicked, as closely as possible, initial rapid-mixing followed by slow-mixing, and finally settlement for a minimum period of fourteen hours, all within a 30 m^3^ circular open tank. Figs 1 and 2 outline the full-scale treatment procedures adopted for each protocol.

### Full-scale operation of Protocol A

The 30 m^3^ treatment tank (reactor) was filled to a maximum level of approximately two-thirds of the total capacity of the tank. The wastewater was then mixed by re-circulation using a gasoline-fueled centrifugal pump so as to obtain a homogenous mix. The established set of bacteriological and physico-chemical parameters measured during the pilot-studies was determined for the wastewater influent from grab samples of approximately 30 to 50 ml. In addition, the COD (mgO_2_/L) of the reactor influent was measured.

The lime slurry was prepared by the addition of hydrated lime to chlorinated drinking water, at a concentration of approximately 20 g/L, in a 200 liter drum, placed on a platform above the reactor tank, directly above the influent pipe. Lime slurry was continuously added to the wastewater, in an attempt to achieve rapid-mixing (with the inflow hose running parallel to the tank wall by means of an ‘elbow-joint’), until the pH level of the circulating wastewater was measured to be greater than, or equal to, 11.4.

Once the target pH level had been reached, the pump was operated continuously at a relatively low revolution rate for approximately 15 minutes, in order to achieve slow-mixing, and consequently to aid flocculation of the reactor contents. The pump was then switched off and the wastewater was left to settle for at least fourteen hours. A small grab sample of the resulting (‘partially treated’) supernatant was removed for analysis using the same set of parameters used to test the untreated wastewater influent. After measuring the depth of sludge in the tank, the supernatant was carefully pumped into a nearby 3.8 m^3^ tank, taking care not to re-suspend the sludge. The contents of this tank were then adjusted to a pH level of between 7 and 8, by the addition of HCl. A final sample of supernatant was removed for analysis as before.

Provided that the effluent had reached a quality considered to be ‘satisfactory’ (defined as having achieved a turbidity level of less than 50 NTU, a pH level of between 6 and 8, and containing fewer than 1,000 thermotolerant coliform CFU per 100 ml), this ‘final effluent’ was then carefully infiltrated into onsite soil trenches. If the effluent quality failed to meet these quality criteria, the entire treatment procedure was repeated before the final effluent was allowed to be infiltrated to the soil.

### Full-scale operation of Protocol B

The process of tank filling was identical to that followed under Protocol A and grab samples of the influent were analyzed for the same parameters prior to treatment.

HCl was then added to the tank contents until the pH level of the circulating wastewater was recorded to be equal to, or lower than, 3.9. Once the target pH level had been reached, the contents were recirculated slowly by pumping for five minutes to ensure that the pH level within the reactor was as homogenous as possible. The pump was then switched off, and the tank contents were left to stand for a minimum period of no less than fourteen hours.

When the depth of sludge in the tank had been measured, the supernatant was carefully pumped (taking care not to re-suspend the limited quantity of sludge that had been produced at this stage) into the nearby smaller tank (3.8 m^3^). The contents of this tank were adjusted to a pH level of between 6 and 7, by the addition of lime slurry, before a grab sample was removed for analysis using the same set of parameters as before. The wastewater at this stage was considered to be ‘partially treated’.

It is perhaps worth noting that, while the addition of HCl, as described above, did not in itself result in coagulation/flocculation, it was considered useful to take advantage of unaided overnight sedimentation before the supernatant was removed for subsequent coagulation/flocculation the next day. The remaining, relatively small quantity of ‘low-pH (disinfected) sludge’ removed from the bottom of the treatment tank in Protocol B was blended with the much larger volume of ‘high-pH sludge’ produced by Protocol A, in order to produce a pH-neutral blend.

A concentrated solution of aluminum sulfate was prepared by dissolving approximately 300 g of the hydrated salt in 1 liter of chlorinated drinking water. Four transparent beakers, each containing 1 liter of wastewater, were used for jar-tests, with the aim of determining the quantity of coagulant needed to achieve adequate sedimentation. This was found to be approximately 100 mg/l. The aluminum sulfate solution was added to each 3.8 m^3^ tank, with manual ‘rapid-mixing’ achieved using a short stirring rod for approximately 5 minutes (flash-mixing), followed by a manual slow-mixing phase of about 15 minutes, using a longer stirring rod (to improve the formation of flocs).

The wastewater was then left to settle for approximately one hour and a grab sample of approximately 30 ml of supernatant (‘final treated effluent’) was tested for the standard parameters, as before. Provided that the effluent had achieved the ‘satisfactory’ quality previously specified under Protocol A, the effluent was carefully infiltrated to the soil. Again, if the quality criteria for satisfactory final effluent had not been met, the coagulation/flocculation procedure, using aluminum sulfate, was repeated. If the effluent quality level had still not met the specified quality standards at this stage, the entire treatment process, including low-pH disinfection and coagulation/flocculation, would have been repeated, but in practice this was never required (Fig 1).

### Sludge treatment

All sludge had been exposed to either the high or low pH environment (that had each demonstrated more than 3-log reduction in levels of thermotolerant coliforms in the supernatant) for at least twenty four hours. Bacterial enumeration of sludge is not possible by membrane filtration as the solids block the pores of the nitrose-cellulose filter. The alternative enumeration method by ‘multiple tube’ (most probable number) was not feasible under the emergency field conditions at the CTC. Therefore, although it can be assumed from the analysis of the supernatant that significant disinfection had been achieved throughout the contact tank during the coagulation-flocculation and subsequent sedimentation stages, the precautionary principle was used in all subsequent handling of the sludge. All sludge was air dried for at least fifteen days and then either carefully placed in infiltration pits (as continues to be common practice for the disposal of fresh, untreated human excreta in many CTC operations around the world) or incinerated along with solid hazardous health-care wastes, as recommended by Gautam et al. [40]. The authors therefore conclude that the hazard of human infection from the sludge was appropriately managed.

### Determination of physico-chemical and bacteriological parameters

The following set of physico-chemical and bacteriological analyses was performed on all Protocol A and Protocol B samples (both during pilot-scale studies and full-scale plant operation). The main aim of all analyses was to determine the degree of reduction in turbidity (NTU) or total suspended solid (TSS), and thermotolerant coliforms (CFU per 100 mL). Measurements of COD concentration were only achieved during full-scale operation of Protocol A. All analyses were undertaken on grab samples, typically 30–50 ml of the wastewater, taken either from the five-liter beakers (pilot-scale trials) or from the full-scale treatment tanks.

### Determination of physico-chemical parameters

Initially, turbidity levels were recorded (as NTU) following a simplified 'turbidity tube' method [44]. This method was later replaced by a spectrophotometric protocol, using a Hach portable turbidimeter (model 2100P), which operated within a wavelength range of 400 to 600 nm. All turbidity data reported here were recorded spectrophotometrically.

Measurement of total suspended solids (as mg/L) was achieved by filtration of the sample through a glass-fiber filter, according to standard methods [45]. As an oven was not available in the field, filters were dried at ambient temperature (normally greater than 30°C) until constant weight was achieved (normally within 48 hours).

pH levels were measured several times during both protocols to minimize the quantity of reagents used to achieve adequate disinfection (and in the case of Protocol A, to ensure effective coagulation and flocculation). The pH level was also frequently measured during the later neutralization phases (both protocols) in order to achieve a final pH level of between 6 and 8. A Palintest Micro 500 pH meter was used for all measurements. pH buffers (7.0 and 4.0) were used for pH meter calibration and pH probes were stored in a saturated KCl solution. In addition, because of the potential for damage to the probe at high and low pH levels, simple pH litmus paper strips were frequently used to verify the pH values obtained.

COD (as mgO_2_/L) was measured using a simplified spectrophotometric field kit (Palintest). The samples were digested at 150°C for two hours in a strong solution of sulfuric acid, in the presence of chromium and silver salts. The tubes were then cooled and the color was measured using the Palintest photometer. Four test kits were used, with a maximum detection level of either 2,000 mg/L or 20,000 mg/L, for analysis of the influent, and either 150 mg/l or 400 mg/l, for analysis of partially, or fully-treated wastewaters.

During the field conditions encountered, the quantities of all chemical reagents used were recorded as accurately as possible during all operations.

### Determination of microbial parameters

Presumptive counts of thermotolerant coliforms were recorded as colony-forming units (CFU) per 100 ml, following membrane filtration through a sterile nitrose-cellulose membrane filter (0.45 μm) (using a DelAgua water-testing kit, sterilized by the production of formaldehyde, formed from burning methanol). Acidic and alkaline samples were washed through the filter with an excess of distilled water for one minute, to ensure that the pH level of the membrane prior to incubation approximated 7. Following filtration, the filters were incubated at 44°C ±1°C for 18 to 24 hours, on sterile absorbent pads, soaked in membrane lauryl sulfate broth (Oxoid). Samples were diluted according to their predicted bacterial counts using de-ionized water. Following incubation, all yellow colonies greater than 2 mm in diameter were enumerated and recorded as CFU of presumptive thermotolerant coliforms per 100 ml of the original sample.

## Results and Discussion

The results from the final eight treatment batches, which were performed when adequate monitoring equipment had become available in the field, are summarized in Table 1. A mean wastewater volume of 12.8 m^3^ was treated in each of the batch processes reported here, six of which were executed according to the low pH procedure (Protocol A), and two of which were executed according to the high pH procedure (Protocol B).

**Table 1:** Comparison of raw and treated wastewater quality from full-scale treatment (batch volumes ranged from 10 to 15 m^3^).

Parameter	Raw wastewater		Treated effluent		Removal
	Mean	Range	Mean	Range	Removal (%)
**Protocol A (low pH): mean results from two batches** *^1^					
Turbidity (NTU)	805	740–870	15	5–26	98.2
Thermotolerant coliforms (CFU per 100 ml)	1.75 x 10^4^	1.7 x 10^4^–1.8 x 10^4^	5	5–5	99.97
COD (mg O_2_/l)	17,080*^2^	-	131	108–154	99.2
Total suspended solids (mg/l)	1,155	980–1330	112	81–143	90.5
**Protocol B (high pH): mean results from six batches** *^1^					
Turbidity (NTU)	430	1200–120	23	2–40	91.3
Thermotolerant coliforms (CFU per 100 ml)	4.98 x 10^4^	1.1 x 10^4^–1.8 x 10^5^	106	20–390	99.52
COD (mg O_2_/l)	17,080*^2^	-	149	134–160	99.1
Total suspended solids (mg/l)	1077	280–4350	38	3–95	92.9
**Summary: mean results from eight batches** *^1^					
Turbidity (NTU)	520	120–1200	21	2–40	93.0
Thermotolerant coliforms (CFU per 100 ml)	4.1 x 10^4^	1.1 x 10^4^–1.8 x 10^5^	81	5–390	99.91
COD (mg O_2_/l)	17,080*^2^	-	144	108–160	99.1
Total suspended solids (mg/l)	1,097	280–4350	57	3–143	92.3

*^1^ Performed when adequate monitoring equipment had become available in the field.

*^2^ Calculated with reference to an average value for untreated wastewater in the absence of quantitatively sufficient data.

### Contaminant removal rates

Treatment by both Protocols A and B achieved an effectively clarified effluent, with a turbidity reduction consistently greater than 80% and a mean reduction of 93% (1.1 log). Mean TSS reduction was 92% (1.1 log). Removal of thermotolerant coliforms was consistently greater than 99.8% (2.7 log), with a mean reduction of 99.9% (3 log). The mean removal of COD (calculated with reference to an average value for untreated wastewater in the absence of sufficient data) was consistently higher than 99% (2 logs).

### Consumption of reagents and levels of residual chemicals

The rate of consumption of chemical reagents during the full-scale treatment operations (when adequate monitoring equipment had become available in the field) is summarized in Table 2. A comparison of the two protocols suggests that, overall, Protocol B was more efficient in terms of the total mass of reagents required to achieve the desired treatment outcome. Protocol B was demonstrated to require on average 1.30 L of HCl per m^3^ of wastewater, compared with 2.25 L HCl per m^3^ wastewater for Protocol A. Additionally, a mean dose of 0.47 kg of Ca(OH)_2_ was required per m^3^ of wastewater for Protocol B, compared with 3.96 kg Ca(OH)_2_ per m^3^ of wastewater for Protocol A. The mean residual aluminum level in the treated effluent from Protocol A was shown to be 0.05 mg per L and 0.07 mg/l for Protocol B. Levels of residual aluminum were never reported to exceed 0.1 mg/l. The mean volume of sludge produced was 6% (vol./vol.).

**Table 2:** Comparison of consumption rates of chemical reagents, residual aluminum and volume of sludge produced from full-scale treatment.

Parameter	Protocol A (low pH)	Protocol B (high pH)
Total volume treated [m^3^]	25	78
Mean concentration of acid used [l/m^3^] = [ml/l]	2.25	1.30
Mean concentration of lime used [kg/m^3^] = [g/l]	3.96	0.47
Mean concentration of AlSO4 used [g/m^3^] = [mg/l]	-	112.5
Residual aluminum concentration in the effluent [g/m^3^] = [mg/l]	0.01	0.07
Approximate sludge volume [m^3^]	0.81	0.71
Approximate sludge volume [%]	5.6	6.2

Operating a novel wastewater treatment plant during the Haitian cholera outbreak presented significant logistical problems, the foremost of which were limited access to adequate supplies of good quality chemical reagents (including lime, alum and hydrochloric acid) and inadequate provision of resources and facilities to support effective operational research. Notwithstanding these obstacles, a novel CTC wastewater treatment and disinfection system was designed and operated successfully, and provided a potentially very useful knowledge-base for further development and application of the technology in other settings.

During the entire operational stage (rather than solely during the final phase reported above), Protocol A demonstrated a greater requirement for chemical reagents than Protocol B (in terms of mass of chemicals to be transported into the field per m^3^ of wastewater to be treated). It is important to note here that variance in the mass of hydrated lime used per unit of wastewater during the execution of Protocol B was much higher than was predicted by initial laboratory tests. This is probably the result of, not only variations in the characteristics of the wastewater between each batch, but also variations in the purity (as percentage weight of CaO) of successive batches of the lime obtained during the challenging circumstances encountered at the time of the study. Additionally, plant operation was undertaken in conjunction with operator training. During initial plant operation (data not reported here) operators were trained to prevent excessive use of reagents that was unnecessary to meet the treatment objectives. The residual levels of aluminum recorded in the treated effluent produced under Protocol B suggest that addition of this coagulant (which is commonly used in drinking water treatment systems), is unlikely to represent a significant risk to the health of human populations living downstream of the treatment system [46].

### Sludge production and treatment

The recorded average volume of produced sludge, at 6% (vol./vol.), was slightly higher than the values recorded in the literature for coagulation/flocculation using hydrated lime and aluminum sulfate [47] [48]. However, sludge volumes slightly in excess of those stated in the literature may be deemed acceptable for this kind of experimental field-work, especially given the practical constraints observed at the time these trials were undertaken. For example, during certain phases of the project, the removal of supernatant was found to have been performed under sub-optimal conditions. This was because it took up to one month to train personnel adequately so as to optimize the process and minimize the sludge volume.

An additional study of the microbiological characteristics of the sludge, before and after its subsequent treatment by solar drying and prior to its incineration or controlled discharge to a protected soil infiltration pit is warranted in the future. This would need to be done using a multiple tube (most probably number) approach rather than the membrane filtration method available to the authors during this study. However, it is important to note that, although pathogens would have been concentrated in the sludge during the treatment process, the evidence suggests that the extreme pH levels to which they were subjected (for an extended period of time) would have resulted in a highly significant reduction in the concentration of viable organisms and that controlled soil infiltration, according to the protocols used elsewhere for untreated CTC wastewaters, constitutes a rational management of the risk of onward human infection.

### Cost analysis

A relatively simple cost analysis demonstrated that labor costs per unit of treated fecal waste for Protocols A and B are roughly equivalent to those of the super-chlorination approach to disinfection, the efficacy of which has been questioned [21] [19] [22] [23]. Moreover, significant financial savings, in relation to reagent costs, may be achieved using the protocols presented here. Further details are provided in the Supporting Information files.

### Conclusions

In light of the recent findings of a panel of experts reporting to the United Nations, the research presented here is timely [49]. The report states that “[…] to prevent introduction of contamination into the local environment, United Nations installations worldwide should treat fecal waste using on-site systems that inactivate pathogens before disposal. These systems should be operated and maintained by trained, qualified […] staff or by local providers with adequate oversight […]” [50]. Although the authors of the report do not prescribe an appropriate microbiological quality standard that might be met by disinfection of the wastewater prior to discharge into the environment, it is interesting to note that concentrations of thermotolerant coliforms in the treated wastewater reported in the study reported here consistently met the WHO bacteriological guideline values for agricultural reuse, i.e., fewer than 1,000 CFU/100 ml [51]. In fact, the quality of the final effluent achieved by both full-scale treatment protocols was consistently higher than the minimum standard initially agreed for disposal by direct infiltration.

The high rate of disinfection achieved using both physico-chemical treatment protocols described here clearly suggests that this innovative technology may be an appropriate and potentially valuable option for the onsite-disinfection of CTC wastewaters generated in the emergency settings encountered during cholera epidemics and potentially may offer a valuable form of wastewater and human excreta disinfection during outbreaks of other infectious diseases. For example, although the Ebola virus is considered to be ‘fragile’ beyond the environment of bodily fluids (including feces), its potential presence in large numbers in the feces of Ebola patients and its relatively low infective dose [52] [53] present a potent hazard to health care workers. The disinfection options presented here may be readily adapted to provide an important *in situ* excreta disinfection step as part of an integrated infection control framework.

More accurate determination of the chemical consumption for both protocols is currently being achieved through laboratory experimentation, but a key finding of the field work reported here, that chemical consumption during the execution of the low pH treatment process (Protocol B) was significantly lower than that during the high pH process (Protocol A), appears to be valid. This consideration potentially has significance for international medical organizations that may wish to use this technology during future disease outbreaks, especially in scenarios where reducing the quantity of chemicals, either purchased locally or imported, may be a high priority.

The evidence available from the published literature suggests that the organism *Vibrio cholerae* is highly likely to respond to extreme levels of pH achieved in the protocols presented here in a similar manner to thermotolerant coliforms (including *Escherichia coli*). However, a future investigation, which compares the behavior of *Escherichia coli* and other commonly used indicator bacteria (such as intestinal enterococci), with that of *Vibrio cholerae* would probably provide valuable additional information to help optimize the treatment technologies presented here.

The engineering priority now must be to monitor these treatment systems under more highly-controlled conditions, in order to refine the treatment processes and validate the data reported here, which were achieved under challenging field conditions. A longer-term challenge for microbial ecologists is to develop a better understanding of how toxigenic strains of *V*. *cholerae* and other excreta-borne pathogens behave in the environmental niches present in wastewater treatment plants [54], but the technology outlined here may have broader application to scenarios in which hygienic management of sludges and wastewaters has to be achieved rapidly and at relatively low-cost. The authors are therefore currently exploring its application to other NTD outbreak settings and to the broader issue of urban excreta management in low-income communities. However, it is essential that those actively involved in WASH operational research should take a multi-disciplinary approach to the issue of controlling disease transmission from human excreta and should avoid the tendency to focus exclusively on infrastructural interventions [11]: those responsible for designing and operating new wastewater treatment technologies in emergency settings should always consider the broader and longer-term public health context of their interventions and should fully evaluate all new technologies within the rational risk management framework of ‘sanitation safety planning’.

## Supporting Information

S1 Text ‘Financial costings recap’. Schematic overview of financial costings.
(PDF)

S2 Text ‘Financial costings detail’. Detailed calculation of financial costings.
(PDF)
