# Supplementary material for: Minimizing the Risk of Disease Transmission in Emergency Settings: Novel In Situ Physico-Chemical Disinfection of Pathogen-Laden Hospital Wastewaters
Source: PLoS Negl Trop Dis. 2015 Jun 25;9(6):e0003776. doi: 10.1371/journal.pntd.0003776 (PMC4482504; doi:10.1371/journal.pntd.0003776)
Supplement: S1 Text — Schematic overview of financial costings. (PDF) [file pntd.0003776.s001.pdf]

Disinfection method →	Superchlorination with HTH (Calcium hypochlorite)	Superchlorination with NaDCC (Klorsept)	High pH physico-chemical treatment	Low pH physico-chemical treatment
Cost for the disinfection of 1 m^3 of pathogen-laden hospital wastewaters [€]	21.6	14.4	6.5	1.3
