# Supplementary material for: Minimizing the Risk of Disease Transmission in Emergency Settings: Novel In Situ Physico-Chemical Disinfection of Pathogen-Laden Hospital Wastewaters
Source: PLoS Negl Trop Dis. 2015 Jun 25;9(6):e0003776. doi: 10.1371/journal.pntd.0003776 (PMC4482504; doi:10.1371/journal.pntd.0003776)
Supplement: S2 Text — Detailed calculation of financial costings. (PDF) [file pntd.0003776.s002.pdf]

Superchlorinatior	Price per unit [€]	Weight [kg]	Dose required to produce 1 litre solution at 2%	Dose required to produce 1 m3 solution at 2%	unit	Price / unit [€]	Price to produce 1 m3 solution at 2%	Quantity product required to disinfect/treat 1m3 wastewater [m3]	Price to disinfect 1m3 wastewater
HTH (Calcium hypochlorite)	3.237	0.45	30	30,000	g	0.00719	216	0.1	**21.6**
NaDCC (Klorsept)			20	20,000	Tablet	0.00720	144	0.1	**14.4**
**Physico-chemical treatment**									
**HCl**									
Low pH	0.15	1						1.3	0.2
High pH	0.15	1						2.25	0.3
**Lime**									
Low pH	1.56	1						0.47	0.7
High pH	1.56	1						3.96	6.2
**AlSO4**									
Low pH	3.232	1						0.112	0.4
High pH	3.232	1						0	0.0
								**Total low pH**	**1.3**
								**Total high pH**	**6.5**
